# Editorial: Language Acquisition in Diverse Linguistic, Social and Cognitive Circumstances

**DOI:** 10.3389/fpsyg.2018.01807

**Published:** 2018-10-24

**Authors:** Maria Garraffa, Maria Teresa Guasti, Theodoros Marinis, Gary Morgan

**Affiliations:** ^1^Psychology Department, Heriot-Watt University, Edinburgh, United Kingdom; ^2^Psychology Department, Università Milano Bicocca, Milan, Italy; ^3^Linguistics Department, University of Konstanz, Konstanz, Germany; ^4^Department of Clinical Language Sciences, School of Psychology and Clinical Language Sciences, University of Reading, Reading, United Kingdom; ^5^Division of Language & Communication Science, City University London, London, United Kingdom

**Keywords:** language acquisition, language pathology, multilingualism, heritage language (HL), second language (L2) acquisition, sign language (SL), syntax, vocabulary acquisition

The language experience of children growing up in linguistically diverse environments is subject to considerable variation both in terms of input quantity and quality and these factors are predictive of future language abilities (e.g. Hart and Risley, 1995). While virtually all typically developing (TD) children acquire language competence, there are large differences in the extent to which vocabulary and higher-level linguistic skills develop, especially in children with atypical language development. This research topic encouraged a debate around the linguistic and environmental factors at play in a set of diverse environments for language acquisition. Language acquisition cannot be investigated without a clear description of the linguistic phenomena that need to be acquired. It is not clear, for example, why some phenomena are acquired later and some earlier; and if differences between children in processing are an effect of differences in competence, or differences in levels of cognitive variables such as non-verbal IQ, working memory, or Executive Functions.

A first theme emerging from the contributions in this research topic is the different trajectories of linguistic phenomena at different developmental stages. Finer aspects of language acquisition do not come from the environment but from maturational changes in early learners. This is the case in Belletti and a study of the children's ability to answer direct object questions. Productions reported by Italian children are non-attested in adults' grammar but are compatible with an immature grammatical system. The study supports the idea that input is not a sufficient variable to explain development and also the outcomes of the study are compatible with developmental trajectories.

A further step in the debate on how to integrate environmental and internal (biological) factors was discussed in a study on German preschool children's comprehension of Relative Clauses (RC). Age modulated the comprehension of Object RCs, with older children being more sensitive to pure grammatical distinctions compared to younger children who were more affected by non-linguistic cues (Adani et al.).

The comprehension of RCs in a trilingual group of children with Cantonese (L1), Mandarin (L2), and English (L3) was investigated by Chan et al. that looked at the effect of limited exposure due to the multilingual environment. Transfer from the head-initial language (English) was reported in the trilingual group in the comprehension of object RCs in Cantonese because of structural overlap and intensive exposure to English. The study points out the importance of identification of vulnerable domains, such as Head noun assignment in object RCs in multilingual Cantonese children acquiring English.

Another group of trilingual children with developmental vulnerability due to scarce input was investigated in a study of vocabulary skills, comparing monolingual, bilingual, and trilingual children (Mieszkowska et al.). For the majority language (English) no difference was found across the three groups. However the minority language was reported as incrementally weaker in both bilingual (reduced expressive vocabulary) and trilingual (reduced expressive and receptive vocabulary) children. The authors suggest that the home language needs to be supported more to achieve a developmental trajectory consistent with the dominant language of the environment.

A well-established pattern in TD children is the greater difficulty in interpreting sentences with pronouns (in particular referential antecedents compared to quantified and full vs. reduced pronouns). Few studies have investigated the interpretation of pronouns in L2 learners. A study on adult L2 learners found that beginners' performance is affected by type of pronoun and antecedent. These results are in line with the grammar of monolingual children, advocating for a general linguistics principle at play in L2 learners (Slabakova et al.).

The authors argue that studies of the developmental trajectory of language development should include the acquisition of different word categories. A significant difference between comprehension and production of both nouns and verbs was reported in a study on child learners of two East African Languages. While the findings were in keeping with previous noun-bias work, making the study cross linguistically valid, a quantitative and qualitative difference was reported. The proportion of spoken verbs correlated with increases in vocabulary size, and with more nouns in the first spoken words and verbs in the comprehended ones (Alcock).

Feijoo et al. questioned the fundamental assumption of semantic bootstrapping in the acquisition of language categories. Investigating child-directed speech input to children under the age of 2;6 they showed that semantic cues alone are not sufficient for word categorization. Rather children need to carry out an analysis of both distributional and semantic cues in the child-directed speech. These results are in line with theories that suggest the need for an integration of multiple cues from different sources in language development.

A second theme was the challenge of how to differentiate multilingual children with slower early language development from multilingual children with developmental language disorder (DLD). While language difficulties in the two populations can look similar, further investigations have reported divergent behaviors between multilingual and atypical populations (Armon-Lotem, 2017). In a study of Dutch children's cognitive and linguistic abilities, Boerma et al. reported that auditory sustained attention mediated the effect of L1 on vocabulary and morphology in both the monolingual and multilingual groups. The study supports the idea that a weak linguistic ability in children with a developmental language disorder (DLD) can be related to an impairment in sustaining attention to auditory stimuli.

Another study on effective tools for differentiating multilingual children and children with DLD used non-word- and sentence repetition as clinical markers (Hamann and Ibrahim). The study showed that the two measures are reliable tools for identification of DLD in multilingual contexts if background information is included. Crucially, both tasks can discriminate multilingual TD children from monolingual children with DLD and multilingual TD children from multilingual children with DLD, with sentence repetition being more affected by language dominance. The study also highlighted that testing in the home language in a heritage context might lead to unreliable classifications.

A further theme was the acquisition of minority languages, including signed languages. Bosma et al. explored the cognitive components ancillary for language acquisition, focusing on the role of verbal working memory (vWM) for the acquisition of phonological regularities in a longitudinal study in a group of Frisian-Dutch bilingual children. The study strongly supported the hypothesis that vWM is an essential component to detect phonological regularities in a task targeting cognates in the two languages.

The role of exposure was addressed in an extensive study (from single words to narratives) of bilingual Polish-English children, focusing on L1 exposure (Haman et al.). The bilingual children scored lower compared to monolinguals in all language domains except discourse, with more pronounced differences in production. Grammar scores were not related to the levels of L1, but were predicted by general cognitive abilities. L2 exposure negatively influenced productive grammar in the L1, suggesting possible L2 transfer effects. Importantly, the authors did not find any evidence that the gap between monolinguals and multilinguals would be fully closed by manipulating L1 input.

Factors affecting children acquiring a minority language should be investigated in interaction with the sociolinguistic context of acquisition. This is the case in a large-scale study on the acquisition of clitic placement in bilectal children (Grohmann et al.). The study revealed early discrimination of enclisis in Cypriot Greek and proclisis in standard Greek, but effects related to the context of acquisition, with proclisis increasing as children enter primary school, advocating for the role of formal education in bilectal settings.

A second study on bilectalism focused on speaker's perception of the two varieties, investigating the hypothesis of a grammatical fluidity in bilectal speakers (Leivada et al.). A variety-judgment task was developed in a large study on monolinguals, bilectals, and bilinguals, including heritage language learners and L1 attriters. The study supported the idea of a different grammatical appreciation in speakers of non-standardized languages (Leivada et al.).

The role of duration of exposure was tested in a study on Deaf children immersed in a dual language input environment (Cantonese and Hong Kong Sign Language, HKSL). The study focused on the acquisition of classifier constructions in HKSL, a structure that emerges later and with cross-linguistics differences between the two languages, in particular verb root and word order. The findings revealed Deaf children's gradual convergence on the adult grammar despite late exposure to HKSL. Evidence of cross-linguistic influence on word order came from the initial adoption of a Cantonese structure. There was also a prolonged period of adherence to the SVO order across all ages (Tang and Li).

Early L2 learners revealed a different performance in reading compared to monolingual children. Vernice and Pagliarini looked at the contribution of morphological awareness to reading in a group of Italian L1 and Arabic-Italian early L2 children. Accuracy in the morphological awareness tasks was a significant predictor of reading fluency. The study highlights the critical role of morphological processing in reading efficiency and suggests that morphological awareness training could improve reading in bilingual students.

Another contribution pointed out the role of the learning scenario in language acquisition, comparing implicit and explicit learning. To assess whether the formation of experience-based expectations is dependent on explicit awareness, Ottl and Behen presented data from an experiment in which gender coding was acquired implicitly. Results showed that participants develop frequency-based expectations comparable to those previously observed in an explicit learning scenario. At the same time, however, the study suggests that expectations surface earlier in the implicit learning scenario.

## Author contributions

MG, MTG, TM, and GM drafted the work and revised it critically for important intellectual content. They did the final approval of the version to be published and are accountable for all aspects of the work in ensuring that questions related to the accuracy or integrity of any part of the work are appropriately investigated and resolved.

### Conflict of interest statement

The authors declare that the research was conducted in the absence of any commercial or financial relationships that could be construed as a potential conflict of interest.
